# A genetic variant rs13293512 in the promoter of let-7 is associated with an increased risk of breast cancer in Chinese women

**DOI:** 10.1042/BSR20182079

**Published:** 2019-05-24

**Authors:** Ruifen Sun, Jianyu Gong, Ju Li, Zhiguo Ruan, Xiaomi Yang, Yongren Zheng, Lili Qing, Xiaoshan He, Jike Jiang, Yanxia Peng, Haijian Zou

**Affiliations:** 1 Yunnan University of Chinese Traditional Medicine, Kunming, Yunnan, China; 2 Department of Forensic Medicine, Kunming Medical University, Kunming, Yunnan, China

**Keywords:** breast cancer, let-7, promoter, polymorphism

## Abstract

Growing evidence has demonstrated that single-nucleotide polymorphisms (SNPs) in the promoter of miRNA may influence individuals’ susceptibility to human diseases. We examined two SNPs rs10877887 and rs13293512 in the promoters of let-7 family to determine if the two SNPs were related to the occurrence of breast cancer (BC). Genotyping of the two SNPs was performed by PCR and restriction fragment length polymorphism analysis or TaqMan assay in 301 BC patients and 310 age matched controls. We found a higher frequency of rs13293512 CC genotype and rs13293512 C allele amongst BC patients (CC vs TT: adjusted odds ratio (OR) = 1.78; 95% CI: 1.14–2.80; *P*=0.012; C vs T: adjusted OR = 1.33; 95% CI: 1.06–1.67; *P=*0.013). Stratification analysis showed that rs13293512 CC genotype was associated with an increased risk of BC in patients with negative estrogen receptor (adjusted OR = 2.39; 95% CI: 1.32–4.30; *P=*0.004), patients with negative progesterone receptor (adjusted OR = 1.92; 95% CI: 1.11–3.33; *P=*0.02), patients with T1-2 stage cancer (adjusted OR = 1.77; 95% CI: 1.07–2.93; *P=*0.03), and patients with N1-3 stage cancer (adjusted OR = 1.89; 95% CI: 1.13–3.17; *P=*0.015). These findings suggest that rs13293512 in the promoter of let-7a-1/let-7f-1/let-7d cluster may be a possible biomarker for the development of BC in Chinese women.

## Introduction

Breast cancer (BC) is the most common invasive cancer and the leading cause of cancer death amongst women worldwide [1]. In China, it accounted for 15% of all new female cancers in 2015 and the mortality rate increased by 59% from 1990–1994 to 2005–2009 [2,3]. It is well known that BC is a multifactor disease, involving in several risk factors, such as tobacco smoking [4], alcohol consumption [5], high fat diet [6], high level of cholesterol [7] as well as genetic factors. However, the exact etiology is still unknown. Therefore, it is of great importance to explore potential genetic biomarkers for effective prevention and treatment of BC.

In 2011, the first association study demonstrated that single-nucleotide polymorphisms (SNPs) in the promoter region of miRNAs may contribute to the susceptibility to cancer [8]. Subsequently, numerous epidemiological investigations revealed that SNPs in the promoter of miRNAs were related to the risk and/or outcome of cancer [9–22]. For instance, Qi and the colleagues found that the rs999885GG genotype in the promoter region of miR-106b-25 cluster had a significantly decreased risk of death for intermediate or advanced hepatocellular carcinoma [21]. Chu et al. found that the rs4705342TC/CC genotypes in the promoter region of miR-143 were associated with a decreased risk of prostate cancer and the T allele can increase protein-binding affinity and reduce transcriptional activity [22].

The lethal-7 (let-7), one of the first two known miRNAs, contains several family members, such as let-7a-1, let-7a-2, let-7a-3, let-7b, let-7c, let-7d, let-7e, let-7f-1, let-7f-2, let-7g, and let-7i [23,24]. Previous studies have shown that let-7 exerts crucial roles in a series of biological processes, including cell mobility, proliferation, apoptosis, migration, and invasion by targetting caspase-3, high mobility group A1, or estrogen receptor-α36 [25–28]. Moreover, let-7 was found to be down-regulated in both BC tissues and cell lines [25,27–32]. All of the above discoveries indicate that let-7 may function as a tumor suppressor in BC.

Let-7 miRNAs have been reported to have tumor suppressive functions. Many known let-7 target genes, such as CCND1, MYC, LIN28, RAS, and HMGA2 are oncogenes involved in cell cycle progression and stemness. In let-7a overexpressed ZR75-1 and MM-231 cells, DICER1 activity was significantly inhibited with decreased miR-208a. The miR-208a-SOX2/β-catenin-LIN28-let-7a-DICER1 can form a feedback loop in the regulation of stem cells renewal [42]. Let-7 on the self-renewal ability of cancer stem-like cells (CSCs) from triple negative breast cancer was also investigated. For example, Sun et al. found that let-7 decreased the tumor formation ability of estrogen-treated breast CSCs and suppressed Wnt signaling, further supporting the previous hypothesis that let-7 decreased the self-renewal ability and contributed to reduce tumor formation ability of stem cells [43].

Recently, two SNPs, rs10877887 and rs13293512, in the promoters of let-7 family have been discovered. Both the SNPs were predicted to influence the binding affinity with transcription factors [13]. Given the potential function, the two SNPs were widely investigated in numerous human diseases, including papillary thyroid carcinoma [12], lung cancer [15], hepatocellular carcinoma [20], intracranial aneurysm [33], major depressive disorder [34], and ischemic stroke [35]. However, the relationship between the two SNPs with BC remains unclear. In the present study, we sought to evaluate the association of the two SNPs with the risk of BC in Chinese women.

## Materials and methods

### Study population

In this hospital-based case-control study, 301 patients with BC were recruited from the hospital over a period of 3 years (between March 2012 and October 2015). The study was approved by the Institutional Review Board of the Yunnan University of Chinese Traditional Medicine and all participants signed informed consent. Each patient was examined and confirmed by histopathological diagnosis. Patients were excluded from the study if they had breast neoplasm and/or inflammation diseases (such as acute and chronic mastitis, papillitis, and breast abscess) and/or an evidence of family history (at least one first- or second-degree relative was diagnosed with BC). Patient information, including age, age of menarche, estrogen receptor (positive or negative), progesterone receptor (positive or negative), human epidermal growth factor receptor-2 (HER2, positive or negative), and TNM classification, was collected from medical record. During the same period of time, 310 healthy volunteers visiting the same hospital for physical examination were selected as controls. Control subjects, living in the same area as the cases, were Chinese Han origin and frequency matched to cases based on age and age at menarche.

### Genotyping

About 2 ml of peripheral blood was collected from each subject in EDTA anti-coagulated tubes at room temperature. Genomic DNA was extracted from blood samples using a phenol–chloroform method. The rs10877887 polymorphism was analyzed by using PCR and restriction fragment length polymorphism (PCR-RFLP) with the restriction enzyme *Fau* I [34]. The rs13293512 polymorphism was analyzed by using a TaqMan SNP genotyping assay [12,33]. For quality control, genotype concordance was verified by DNA sequencing.

### Statistical analysis

Statistical analysis was done using SPSS version 13.0 software (SPSS Chicago, IL, U.S.A.). The chi-square test was used to determine if the observed frequencies of rs10877887 and rs13293512 genotypes were in accordance with Hardy-Weinberg equilibrium (HWE). Continuous variables were expressed as mean ± S.D., and Student’s *t* test was used to find the difference between cases and controls. Odds ratios (ORs) and 95% CIs were calculated to evaluate the association between rs10877887 and rs13293512 polymorphisms and BC risk. Unconditional logistic regression analysis was performed after adjustment for age and age at menarche. All *P*-values were two-tailed, and significance level was defined as *P*<0.05.

## Results

The demographic and clinical data of BC patients and controls are summarized in Table 1. The mean age of BC patients was 51.8 years, and the mean age of controls was 50.4 years. The mean age at menarche for BC patients was 14.0 years, and the mean age at menarche for controls was 14.1 years. There were no differences according to age and age at menarche between cases and controls, with *P*-values of 0.13 and 0.86, respectively. Amongst the patients, 57.8% had positive estrogen receptor and 49.5% had positive progesterone receptor. TNM classification revealed T1-2 in 67.4%, N0 in 41.5%, and M0 in 98.7% of patients.

**Table 1:** Characteristics of the study population

Variables	Patients with breast cancer (*n=* 301)	Controls (*n=* 310)	*P*-value
Age (years, mean ± S.D.)	51.8 ± 10.0	50.4 ± 11.9	0.13
Age at menarche (years, mean ± S.D.)	14.0 ± 1.5	14.1 ± 1.6	0.86
Estrogen receptor (%)			
Positive	174 (57.8)		
Negative	127 (42.2)		
Progesterone receptor (%)			
Positive	149 (49.5)		
Negative	152 (50.5)		
Human epidermal growth factor receptor-2 (%)			
Positive	72 (23.9)		
Negative	229 (76.1)		
Primary tumor (T, %)			
T1-2	203 (67.4)		
T3-4	98 (32.6)		
Regional lymph nodes (N, %)			
N0	125 (41.5)		
N1-3	176 (58.5)		
Distant metastasis (M, %)			
M0	297 (98.7)		
M1	4 (1.3)		

The genotype frequencies of rs10877887 and rs13293512 were in line with HWE amongst healthy controls (*P=*0.65 and 0.75, respectively). The differences of the two polymorphisms between BC patients and controls were presented in Table 2. A higher frequency of rs13293512 CC genotype (24.9 vs 17.1%; adjusted OR = 1.78; 95% CI: 1.14–2.80; *P=*0.012) and rs13293512 C allele (47.8 vs 40.8%; adjusted OR = 1.33; 95% CI: 1.06–1.67; *P=*0.013) were observed amongst BC patients than controls. No significant difference of rs10877887 was found between BC patients and controls.

**Table 2:** Association between rs10877887 and rs13293512 polymorphisms and BC risk

Polymorphisms	Controls, *n=* 310 (%)	BC patients, *n=* 301 (%)	Adjusted OR (95% CI)^†^	*P*-value^†^
rs10877887				
Genotypes				
TT	140 (45.2)	144 (47.8)	1.00	
CT	134 (43.2)	117 (38.9)	0.85 (0.61–1.20)	0.35
CC	36 (11.6)	40 (13.3)	1.10 (0.66–1.83)	0.72
Alleles				
T	414 (66.8)	405 (67.3)	1.00	
C	206 (33.2)	197 (32.7)	0.99 (0.78–1.25)	0.90
rs13293512				
Genotypes				
TT	110 (35.5)	88 (29.2)	1.00	
CT	147 (47.4)	138 (45.8)	1.20 (0.83–1.73)	0.33
CC	53 (17.1)	75 (24.9)	1.78 (1.14–2.80)	0.012
Alleles				
T	367 (59.2)	314 (52.2)	1.00	
C	253 (40.8)	288 (47.8)	1.33 (1.06–1.67)	0.013

† Adjusted by age and age at menarche.

When we stratified the data by estrogen receptor, progesterone receptor, and HER2, the frequency of rs13293512 CC genotype was significantly increased in patients with negative estrogen receptor (adjusted OR = 2.39; 95% CI: 1.32–4.30; *P=*0.004) and patients with negative progesterone receptor (adjusted odds ratio (OR) = 1.92; 95% CI: 1.11–3.33; *P=*0.02) and patients with negative HER2 (adjusted OR = 1.79; 95% CI: 1.10–2.91; *P=*0.018) compared with respective controls. When the data were stratified based on TNM classification, we found a significant association for rs13293512 CC genotype in patients with T1-2 stage cancer (adjusted OR = 1.77; 95% CI: 1.07–2.93; *P=*0.03). Such an association was also observed in patients with N1-3 stage cancer (adjusted OR = 1.89; 95% CI: 1.13–3.17; *P=*0.015) (Table 3). After stratification analysis of rs10877887 with clinical features, no significant association was noticed (Table 4). Furthermore, we did combined analysis of rs10877887 and rs13293512 with BC risk. No significant difference of combined genotypes was observed between cases and controls (Table 5).

**Table 3:** Stratification analysis of rs13293512 polymorphism with BC risk

Genotypes	Controls, n (%)	Cases, n (%)		Case I vs controls		Case II vs controls	
		Case I	Case II	Adjusted OR (95% CI)^†^	*P-* value^†^	Adjusted OR (95% CI)^†^	*P-* value^†^
		ER (+)	ER (-)				
TT	110 (35.5)	57 (32.8)	31 (24.4)	1.00		1.00	
CT	147 (47.4)	77 (44.2)	61 (48.0)	1.04 (0.68–1.60)	0.84	1.48 (0.90–2.43)	0.12
CC	53 (17.1)	40 (23.0)	35 (27.6)	1.47 (0.87-2.48)	0.15	2.39 (1.32–4.30)	0.004
		PR (+)	PR (-)				
TT	110 (35.5)	47 (31.5)	41 (27.0)	1.00		1.00	
CT	147 (47.4)	65 (43.6)	73 (48.0)	1.06 (0.68–1.67)	0.79	1.34 (0.85–2.12)	0.20
CC	53 (17.1)	37 (24.8)	38 (25.0)	1.65 (0.96–2.85)	0.07	1.92 (1.11–3.33)	0.02
		HER2 (+)	HER2 (-)				
TT	110 (35.5)	21 (29.2)	67 (29.3)	1.00		1.00	
CT	147 (47.4)	33 (45.8)	105 (45.9)	1.19 (0.65–2.17)	0.57	1.20 (0.81–1.79)	0.37
CC	53 (17.1)	18 (25.0)	57 (24.9)	1.68 (0.81–3.45)	0.16	1.79 (1.10–2.91)	0.018
		T1-2	T3-4				
TT	110 (35.5)	57 (28.1)	31 (31.6)	1.00		1.00	
CT	147 (47.4)	97 (47.8)	41 (41.8)	1.29 (0.85–1.95)	0.22	1.01 (0.59–1.72)	0.97
CC	53 (17.1)	49 (24.1)	26 (26.5)	1.77 (1.07–2.93)	0.03	1.80 (0.96–3.35)	0.07
		N0	N1-3				
TT	110 (35.5)	34 (27.2)	54 (30.7)	1.00		1.00	
CT	147 (47.4)	64 (51.2)	74 (42.0)	1.43 (0.88–2.33)	0.15	1.05 (0.68–1.62)	0.82
CC	53 (17.1)	27 (21.6)	48 (27.3)	1.64 (0.89–2.99)	0.11	1.89 (1.13–3.17)	0.015

ER, estrogen receptor; PR, progesterone receptor.

† Adjusted by age and age at menarche.

**Table 4:** Stratification analysis of rs10877887 polymorphism with BC risk

Genotypes	Controls, n (%)	Cases, n (%)		Case I vs controls		Case II vs controls	
		Case I	Case II	Adjusted OR (95% CI)^†^	*P*- value^†^	Adjusted OR (95% CI)^†^	*P-* value^†^
		ER (+)	ER (-)				
TT	140 (45.2)	84 (48.3)	60 (47.2)	1.00		1.00	
CT	134 (43.2)	69 (39.7)	48 (37.8)	0.87 (0.58–1.29)	0.48	0.84 (0.54–1.31)	0.44
CC	36 (11.6)	21 (12.1)	19 (15.0)	1.00 (0.54–1.83)	0.99	1.24 (0.65–2.34)	0.52
		PR (+)	PR (-)				
TT	140 (45.2)	73 (49.0)	71 (46.7)	1.00		1.00	
CT	134 (43.2)	61 (40.9)	56 (36.8)	0.89 (0.58–1.34)	0.57	0.82 (0.54–1.26)	0.37
CC	36 (11.6)	15 (10.1)	25 (16.4)	0.81 (0.41–1.58)	0.53	1.41 (0.78–2.54)	0.26
		HER2 (+)	HER2 (-)				
TT	140 (45.2)	32 (44.4)	112 (48.9)	1.00		1.00	
CT	134 (43.2)	33 (45.8)	84 (36.7)	1.05 (0.61–1.80)	0.87	0.80 (0.55–1.15)	0.23
CC	36 (11.6)	7 (9.7)	33 (14.4)	0.87 (0.35–2.16)	0.76	1.17 (0.68–2.00)	0.57
		T1-2	T3-4				
TT	140 (45.2)	98 (48.3)	46 (46.9)	1.00		1.00	
CT	134 (43.2)	80 (39.4)	37 (37.8)	0.85 (0.58–1.24)	0.40	0.86 (0.53–1.42)	0.56
CC	36 (11.6)	25 (12.3)	15 (15.3)	1.04 (0.58–1.85)	0.91	1.27 (0.64–2.55)	0.50
		N0	N1-3				
TT	140 (45.2)	61 (48.8)	83 (47.2)	1.00		1.00	
CT	134 (43.2)	49 (39.2)	68 (38.6)	0.84 (0.54–1.31)	0.45	0.87 (0.58–1.30)	0.49
CC	36 (11.6)	15 (12.0)	25 (14.2)	0.96 (0.49–1.89)	0.90	1.19 (0.66–2.12)	0.56

ER, estrogen receptor; PR, progesterone receptor.

† Adjusted by age and age at menarche.

**Table 5:** Combined analysis of rs10877887 and rs13293512 with BC risk

Combined genotypes	Controls (%)	BC patients (%)	OR (95% CI)	*P*-value
rs10877887TT + rs13293512TT	53 (17.1)	43 (14.3)	1.00	
rs10877887TT + rs13293512CC/CT	87 (28.1)	101 (33.6)	1.43 (0.87–2.35)	0.15
rs10877887CC/CT + rs13293512TT	57 (18.4)	45 (15.0)	0.97 (0.56–1.71)	0.92
rs10877887CC/CT + rs13293512CC/CT	113 (36.5)	112 (37.2)	1.22 (0.76–1.97)	0.41

## Discussion

To our knowledge, it is the first time to explore the potential correlation of SNPs rs10877887 and rs13293512 in the promoters of let-7 family to the risk of BC. We found a significance of rs13293512 CC genotype increasing the risk of BC. Stratification analysis yielded a similar association of rs13293512 CC genotype in patients with negative estrogen receptor or patients with negative progesterone receptor, or patients with T1-2 stage cancer, or patients with advanced cancer. However, no association with BC risk was observed for SNP rs10877887 in both overall and stratification analyses. Since age and age at menarche may affect ORs, we calculated ORs adjusted for age and age at menarche. These findings suggest that rs13293512 CC genotype might contribute to the carcinogenesis of BC in Chinese women.

Let-7 is highly conserved well-established promoters of terminal differentiation that are expressed in healthy adult tissues and frequently repressed in cancer cells. It is widely accepted that let-7 is down-regulated in BC, playing an important role as a tumor suppressor [25,27–32]. The down-expression can induce epirubicin resistance by enhancing cellular apoptosis [29]. Overexpression of let-7 in BC cells can decrease cell proliferation, colony formation, migration and invasion, and vice versa [25,31,32]. Additionally, let-7 family has been reported to limit the numbers of stem cells in normal and cancerous tissue samples, aiding in the maintenance of the differentiation of stem cells and CSCs, thus inhibiting tumor progression. Given the key role of let-7 in BC, let-7 related epidemiological studies are of great interest in the BC world. Previous work focussed on SNPs in the 3′-UTR of the target gene of let-7 [36–39]. For example, Huang et al. reported an SNP rs712 within the binding site of *KRAS* 3′-UTR was associated with regional lymph nodes metastasis of BC [39]. Jiang et al. reported that an SNP rs7963551 C allele in the 3′-UTR of *RAD52* was associated with a reduced BC risk [38].

Recently, it has been identified that SNPs in the promoter region of miRNA were associated with the risk and/or outcome of BC [40,41]. In the present study, we found that rs13293512 CC genotype in the promoter of let-7a-1/let-7f-1/let-7d cluster had a 1.78-fold increased risk of BC. In agreement with our result, Sima et al. reported that rs13293512CT genotype was associated with a 1.43-fold increased risk of developing intracranial aneurysm [33]. Liang et al. reported that rs13293512 CC genotype had a 1.83-fold increased risk of major depressive disorder [34]. Although the precise biological mechanism of SNP rs13293512 contributing to BC development remains unknown, growing evidence demonstrates that SNPs in the promoter of miRNA may affect the expression of mature miRNA and ultimately influence individuals’ susceptibility to human diseases [17,33]. Based on this background, it is reasonable to speculate that patients harboring rs13293512 CC genotype may display a lower level of let-7 expression, resulting in a higher risk to develop BC. Further studies are therefore needed to clarify the biological function of rs13293512 in BC tumorigenesis.

In the subgroup analysis, we found with interest that the rs13293512 CC genotype increased early T classification but late N classification. It is difficult to explain the exact reason for this phenomenon. Some possibilities may be taken into consideration. The sample size in stratification analysis is very limited, with only 203 patients with T1-2 and 176 patients with N1-3 tumors, which may have insufficient power to test the real effects. Moreover, *P* value in T1-2 comparison is 0.03, and we cannot rule out the possibility that the result may occur by chance. Further studies with larger sample size are warranted to confirm these results.

In the present study, we also investigated the relationship of rs10877887 with BC risk. However, we did not find any difference of rs10877887 between cases and controls. Inconsistent with our result, SNP rs10877887 CT/CC genotypes were reported to be a risk factor for lung cancer [15] and hepatocellular carcinoma [20]. In contrast, a reduced risk of rs10877887 CT/CC genotypes was reported in patients with papillary thyroid carcinoma [12]. There is no clear explanation for the conflicting results regarding the impact of SNP rs10877887 on different cancer types. Different genetic backgrounds and/or environmental factors may imply an increasing or decreasing risk for various types of cancers. Further association studies in different cancer types and/or different populations are valuable to verify this result.

Since the study population was limited to Chinese Han women, the positive effect cannot be directly extrapolated to other ethnicities. The follow-up data were not collected in the study design, and thus, we cannot evaluate the effect of the rs13293512 CC genotype on overall outcome of BC. Moreover, the samples are relatively small in the present study, especially in subgroup analysis, which provides limited power to obtain precise results. Future investigations with larger samples in diverse ethnic groups are therefore needed to validate our findings. Although there were some limitations in the present study, our data provides a first indication that rs13293512 in the promoter of let-7a-1/let-7f-1/let-7d cluster may be a possible biomarker for the development of BC in Chinese women.

In conclusion, our assessment of the influence of rs10877887 and rs13293512 on BC showed an increased risk for individuals carrying rs13293512 CC genotype.

## Abbreviations

BC: breast cancer
CSC: cancer stem cell
HER2: human epidermal growth factor receptor-2
HWE: Hardy-Weinberg equilibrium
OR: odds ratio
SNP: single-nucleotide polymorphism
